# Comprehensive neurosurgical and visceral surgical therapy of retroperitoneal nerve tumors: a descriptive and retrospective analysis

**DOI:** 10.1186/s12957-024-03557-5

**Published:** 2024-10-22

**Authors:** Martin Petkov, Marko Kornmann, Ute Marlies Bäzner, Lena Minzenmay, Andrej Pala, Maria Teresa Pedro, Christian Rainer Wirtz, Gregor Antoniadis

**Affiliations:** 1 Bezirkskrankenhaus Günzburg, Peripheral Nerve Surgery Unit, University of Ulm, Lindenallee 2, 89312 Günzburg, Germany; 2 Department of General and Visceral Surgery, University of Ulm, Albert-Einstein-Allee 23, 89081 Ulm, Germany; 3 Department of Neurosurgery, University of Ulm, Albert-Einstein-Allee 23, 89081 Ulm, Germany

**Keywords:** BPNST, MPNST, Nerve, Tumor, Retroperitoneum, Visceral, Peripheral, Surgery

## Abstract

Nerve tumors in the retroperitoneal space are a rarity. Radical surgery according to soft tissue tumors can lead to persistent pain and neurological deficits. This study aims to evaluate clinical outcomes of patients treated by a visceral- / neurosurgical approach. 33 patients with a retroperitoneal nerve tumor underwent surgery between 01/2002 and 12/2022 at our department. A visceral surgeon provided access to the retroperitoneal space, followed by micro-neurosurgical tumor preparation under neuromonitoring. Clinical examination and MRI were performed 12 weeks after surgery and further 3 months (WHO grade > 1) or 12 months (WHO grade 1). Further examinations were based on MRI findings and residual symptoms with median follow-up time of 24 months. One patient was treated for two distinct masses resulting in a total of 34 histological findings. Schwannomas (*n* = 15; 44.1%) and neurofibromas (*n* = 10; 29.4%) were the most common tumors. Long-term improvements were noted in radicular pain (15/18 patients; 83.3%), motor deficits (7/16 patients; 43.8%), abdominal discomfort and pain (5/7 patients; 71.4%). Recurrences were observed in 3/33 (9,1%) patients. This study represents the largest series of retroperitoneal BPNSTs treated with microsurgical techniques. Prospective multicenter studies are warranted to establish standardized treatment guidelines.

## Introduction

Primary retroperitoneal tumors are rare, representing approximately 0.1–0.2% of all neoplasms in the human body [1]. Typical symptoms can vary depending on their size and location. It may include abdominal discomfort or swelling, bowel or bladder disorders, weight loss as well as nerve-related symptoms [2]. Retroperitoneal nerve tumors represent only 10 to 20% of all primary retroperitoneal masses. In contrast to neoplasms arising from the mesodermal system, these tumors are typically slow-growing and non-cancerous with most common types being benign peripheral nerve sheath tumors (BPNSTs) such as neurofibromas and schwannomas [3]. While mostly being benign, there are also infiltrative and malignant entities such as malignant peripheral nerve sheath tumors (MPNSTs), that can occur spontaneously or associated with neurofibromatosis type 1 (NF1) [4]. Due to the proximity to important neuronal structures, patients can present with symptoms such as radicular pain, numbness or weakness in the legs caused by compression from the tumor [5]. However, symptoms are often unspecific and can be similar to those of many other conditions. Due to the variability of involved peritumoral structures, the selection of the optimal surgical approach is often challenging [3]. Tumors in this area are operated by various medical specialties including gynecology, general surgery and urology which further reduces the routine and average number per surgeon. In cases of nerve tumors, this could lead to suboptimal outcome like postoperative neurological malfunction [5]. A monodisciplinary neurosurgical approach could also lead to complications due to less familiarity with anatomical structures and access routes within the retroperitoneal space. In the presented study, a visceral surgeon created a safe access route to the retroperitoneal space, while the microsurgical preparation of the tumor was performed by a neurosurgeon under neuromonitoring. Involving both disciplines as a standard, we evaluated all patients who underwent surgery for retroperitoneal nerve tumors between 2002 and 2022 at our neurosurgical department. Knowing the limited data on these rare cases, our study aimed to assess the effectiveness of the chosen approach for patient’s clinical outcome.

## Methods

### Study framework and clinical pathway

This retrospective study included 33 patients treated over a period of 240 months between 01/2002 and 12/2022 at our department. Relevant information was extracted and evaluated from written and digital patient records for all patients. In cases where imaging findings were not available, they were obtained with the consent of the patients after consultation with radiological practices. All included patients were from the German catchment area. Depending on symptoms and previous diagnostic findings, various imaging was performed, including X-ray, abdominal ultrasound, and computed tomography. The initial suspected diagnosis made outside our department was not documented for all patients. A direct referral from the general practitioner without prior external assessment was documented in only 3 / 33 patients. However, prior to surgery, each patient underwent at least one outpatient consultation at our department and MRI imaging of the tumor (including contrast agent) that was no older than six months. In case of suspected malignant peripheral nerve sheath tumors (MPNSTs), a preoperative ^18^F-FDG PET-CT imaging was supplemented [6]. In all patients with an unclear retroperitoneal mass, surgical intervention was recommended for diagnostic confirmation and treatment. One patient opted against surgery and therefore was not included in the study. Based on the suspected histology and in accordance with our local tumor board’s recommendations, preoperative imaging was discussed for each patient to exactly plan the proper surgical approach. A correct preoperative diagnosis based on the available imaging was achieved in 24 out of 34 cases (70.6%). It is well known that a reliable distinction between neurofibromas, schwannomas, and hybrid tumors is not possible based solely on MR imaging [7, 8]. Assuming no further differentiation of these tumors, all of which were treated with nerve-sparing enucleation (Table 1), the correct preoperative diagnosis was made in 30 out of 34 cases (88.2%), as all BPNSTs were recognized as such preoperatively.

### Baseline patient information

As part of the study, gender, age and presence of NF1 / NF2 were collected retrospectively. During hospitalization, medical history, clinical and neurological examination were conducted to evaluate symptoms, such as radicular and abdominal pain or discomfort, bladder or bowel dysfunction as well as sensory and motor deficits in the lower extremities. In the latter, a further differentiation was made with respect to the documented Janda muscle strength score of the affected muscle groups [9]. Motor deficits were classified as mild (4/5), moderate (3/5) and severe (2/5 or less).

### Intraoperative setting and surgical technique

The surgical team consisted of a visceral surgeon and a neurosurgeon during the entire time of surgery. The operating neurosurgeon remained the same throughout the entire period. In only 3 out of all procedures, the visceral surgeon differed from surgery to surgery. Firstly, a safe access was created depending on the anatomical location of the tumor. In most cases a lower median laparotomy (10–12 cm length) was chosen. In some cases, a right or left lateral (pararectal) extraperitoneal access was used. In cases of a high dorsal tumor extension, the choice was made either for a dorsal approach through a flank incision or a two-stage procedure involving both anterior and dorsal access. Subsequently, the microsurgical preparation of the tumor was performed under neuromonitoring. In cases of circumscribed BPNSTs, a non-responding fascicle was located by electrostimulation and the tumor content was carefully removed after entering the pseudocapsule. In cases of unresectable benign tumors such as perineuriomas and intraneural ganglion cysts, a biopsy was performed after electrostimulation to preserve nerve integrity. In cases of MPNSTs with advanced tumor, presence of NF1 and clear intraoperative findings, an extensive resection including the tumor capsule was performed. The visceral surgeon was present throughout the entire operation in cases of uncontrollable bleeding from iliac vessels or surrounding organs and to assure proper close of the abdominal wall.

### Postoperative monitoring and follow-up assessments

After surgery, the mentioned preoperative findings were assessed postoperatively during hospitalization, and then for benign tumors again at 3 months and 1 year after discharge (with renewed MRI). Further follow-up time intervals differed based on clinical and imaging findings. In cases of MPNSTs, an adjuvant chemoradiotherapy and 3-month follow-up were initiated after evaluation by our interdisciplinary tumor board. The median documented follow-up time of all patients was 24 months.

## Results

### Patient demographics and histopathological results

The study included 33 patients, of which 13 patients (39.4%) were male and 20 patients (60.6%) female. The average age was 41.5 years with a range of 13 to 78 years. Tumor size ranged from 1.2 to 9.6 cm in diameter. The histological examination at the Institute of Neuropathology (Table 1) is shown below.

**Table 1:** Histopathological findings and selected surgical technique

Histology (*n* = 34)	Proportion	Surgical technique
Schwannomma WHO I°	44.1% (15/34)	Enucleation
Neurofibroma WHO I°	29.4% (10/34)	Enucleation
Perineurioma WHO I°	8.8% (3/34)	Epineuriotomy and biopsy
Schwannoma/Neurofibroma (Hybrid tumor WHO I°)	5.9% (2/34)	Enucleation
MPNST WHO IV°	5.9% (2/34)	Resection including capsule
Ganglioneuroma WHO I°	2.9% (1/34)	Enucleation
Intraneural ganglion cyst WHO I°	2.9% (1/34)	Open biopsy

A total of 36 surgeries were performed on 33 patients for 34 different retroperitoneal nerve tumors. In one case with large intra- and extraforaminal schwannoma, a two-stage resection was performed. In another patient, 2 surgeries were performed for different retroperitoneal nerve tumors (neurofibroma and MPNST). One patient suffering from a neurofibroma underwent a second surgery after 24 months for recurrence. In 30/36 (83.3%) surgeries, an anterior approach via median laparotomy was chosen. A dorsal approach via flank incision was indicated in 4/36 (11.1%), a lateral pararectal incision in 2/36 (5.6%) cases. While none of our patients suffered from a known NF2, 4/33 patients (12,1%) had a known NF1: in the case of 2 neurofibromas and 2 MPNSTs.

### Preoperative symptoms and neurological deficits

The majority of patients reported impairment in daily life because of various complaints due to the mass. Radicular pain with radiation to the lower extremities was reported by 18 (54.5%) patients, while sensory deficits were reported by 10 (30.3%) patients. Mild motor deficits were often already present. In 11 patients (33.3%), a muscle group or multiple muscle groups had a 4/5 rating according to Janda scale [9]. Moderate paresis (grade 3/5) was present in 3 (9.1%) patients and severe paresis (grade 2/5 or less) in 2 (6.1%) of the collective. Abdominal discomfort, manifested as loss of appetite, bloating, or localized abdominal pain without dysfunction, was reported by 7 (21,2%) patients. 4 patients (12.1%) had bladder or bowel dysfunction. One patient reported burning dysesthesias as sign of neuropathic pain and another patient had a DJ catheter placed due to impending ureteral compression. In 5 patients (15,2%), the retroperitoneal mass was incidentally detected. These patients had no complaints associated with the tumor.

### Surgical setting

Considering all documented data, the mean surgical time was 211 min with a mean blood loss of 284 ml. In one case of significant blood loss due to increased tumor bleeding, a blood transfusion was administered.

**Fig. 1:**
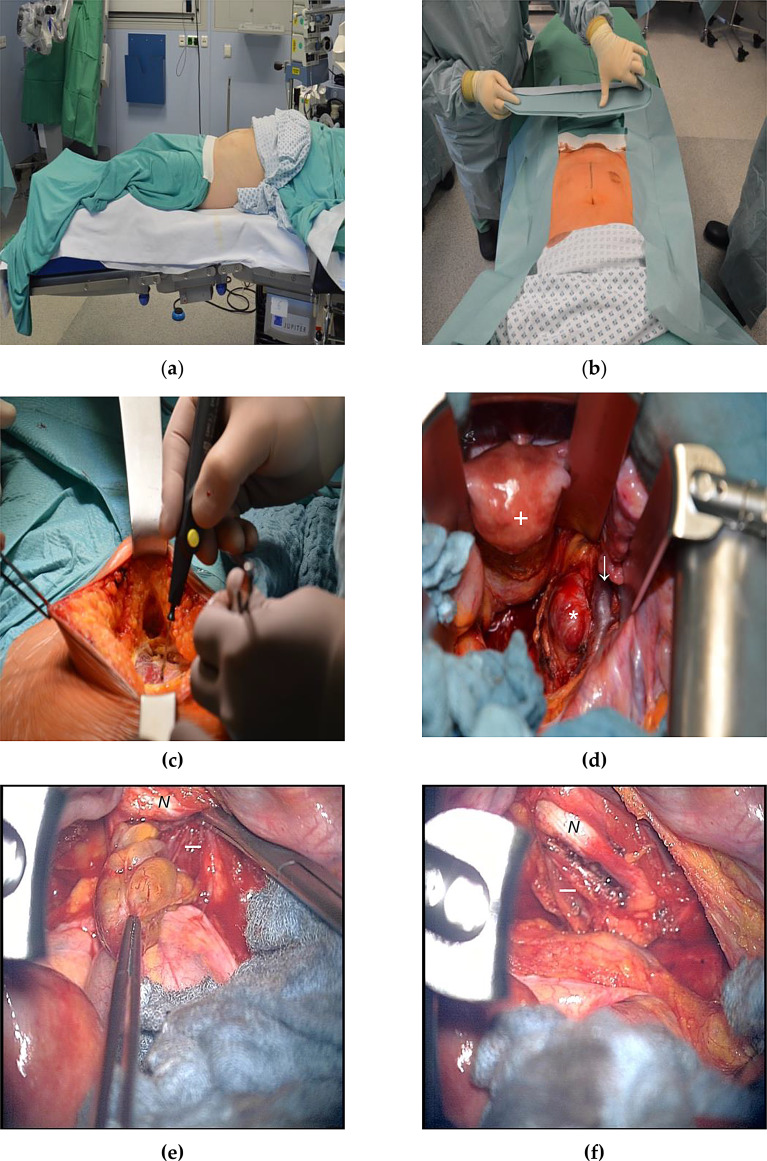
The patient is in supine position (**a**). After marking the skin incision (**b**), a median lower abdominal laparotomy is performed (**c**) followed by careful depth preparation via transperitoneal approach to expose the tumor (*) while preserving the iliac vessels (↓) and uterus (+), which are in close relation to the mass (**d**). After exposing the pseudocapsule (-), an optimal entry point into the tumor is identified using electrostimulation for subsequent enucleation with preservation of the sciatic nerve (*N*); (**e**). At the end of the operation, the remaining fascicle-bearing capsule is left intact (**f**)

A typical surgical procedure is illustrated in Fig. 1. In the following case, a 58-year-old female patient suffered from a schwannoma of the right sciatic nerve. Preoperatively, the patient reported disruptive pressure pain in the right groin area and weakness of the dorsal extension of the right foot (2/5).

**Fig. 2:**
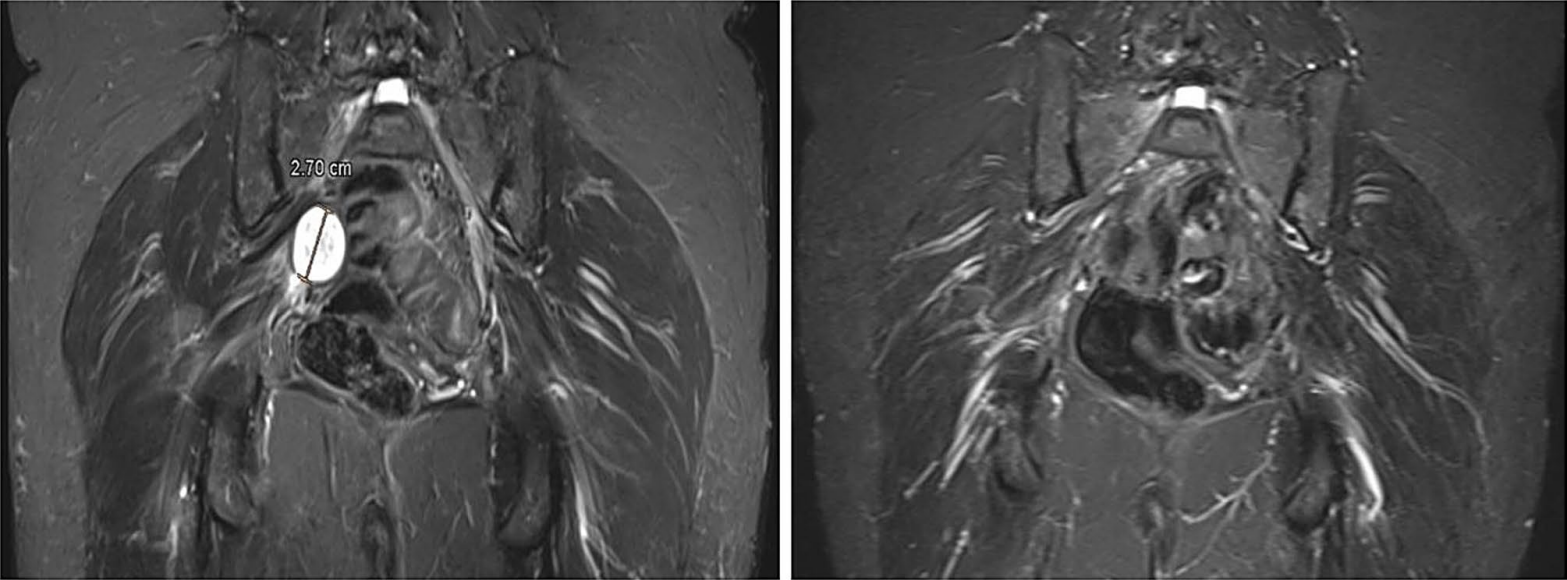
Pre- and first postoperative MRI after 3 months

According to our protocol, an outpatient follow-up examination was performed 3 months postoperatively. An absence of pain and relevant improvement of the paresis (4/5 according to Janda) could be observed. The MRI showed no recurrence (Fig. 2).

### Outcome in the postoperative inpatient and outpatient setting

**Fig. 3:**
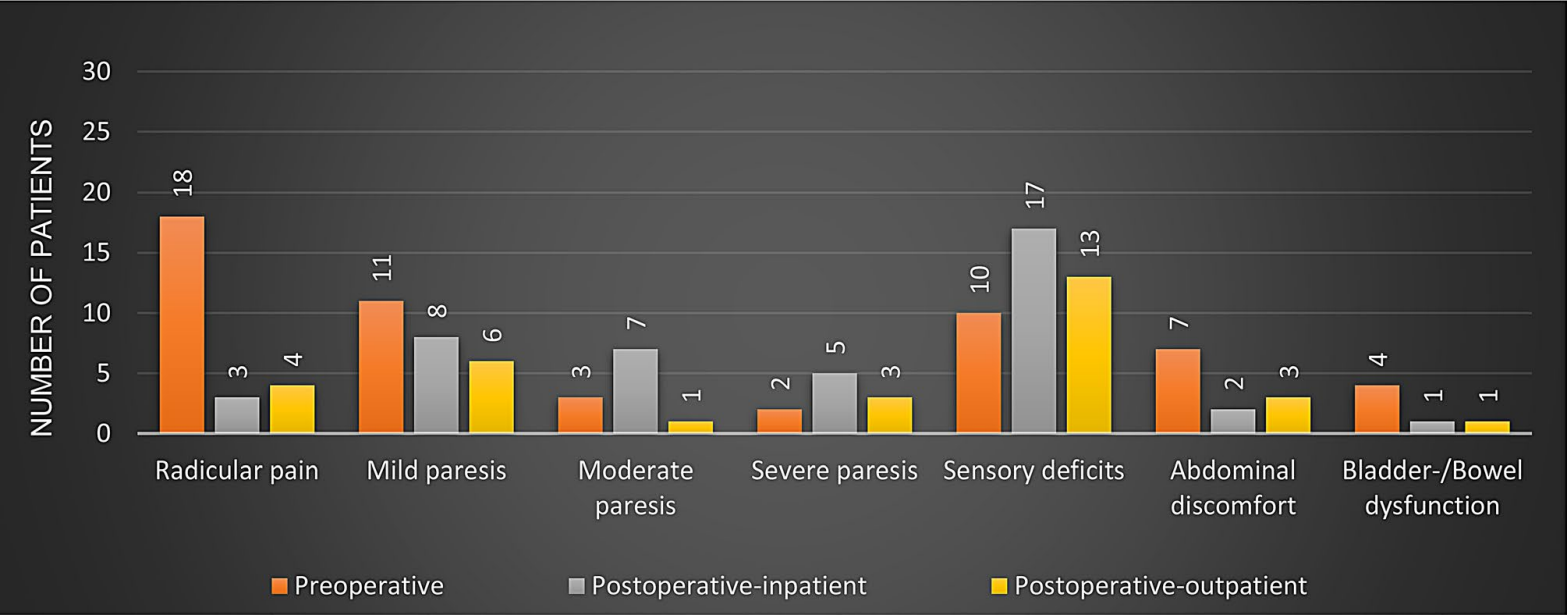
Symptom advancement in patients pre- and post-surgery (median follow up 24 months)

The pre- / postoperative clinical status of the treated patients is summarized in Fig. 3. In early postoperative period, most patients showed relief of radicular pain symptoms. Only 3/33 (9.1%) reported persistent radicular complaints which were well manageable with oral pain medication and allowed for proper mobilization.

Both patients requiring adjuvant treatment with chemoradiotherapy due to a malignant diagnosis (MPNST) reported relevant pain relief. One of them had preexisting severe paresis in the lower extremities and exhibited a motor deterioration postoperatively due to resection of the donor nerve root for prognostic purposes. The other patient with MPNST reported rapid recurring pain after adjuvant treatment. In this case, a recurrence of the tumor was found on the right extraspinal side, with reference to L2-S1. Given the extensive tumor, we advised against a second resection. The patient underwent a hemipelvectomy in an orthopedic university hospital and, according to the report, died promptly due to re-bleeding and multiple revisions. Regarding sensory deficits, a temporary increase from 10 to 17 out of 33 patients (51.2%) was initially observed in the inpatient setting regressing to 13 patients (39.4%) during the subsequent outpatient follow-ups. It should be noted that the reported hypoesthesia area was much smaller postoperatively (mostly residual or new hypoesthesia of the toes compared to manifest sensory loss in a dermatome). All shown results for the late follow-up (outpatient) data are based on the most recently documented report of our neurosurgical ambulance (8th of February, 2024).

## Discussion

Nerve tumors are rarely found in the retroperitoneal space and represent a minority among primary masses in this location. Due to non-existing or non-specific symptoms, heterogeneous imaging findings and anatomical proximity to important neuronal, abdominal and vascular structures, nerve-sparing treatment often seems challenging. At the same time, the rate of initially incorrect diagnoses of peripheral nerve tumors is high. In a retrospective analysis by Uerschels et al., 38 out of 85 patients with a BPNST were initially misdiagnosed, and 26.7% of these patients subsequently underwent inappropriate surgical treatment, resulting in severe neurological deficits [10]. As described previously, current literature on retroperitoneal nerve tumors regarding surgical treatment and neurological outcome is limited due to low case numbers, treatment by different medical fields and lack of multicentric studies [5]. Consideration of long term neurological and pain related symptoms is rare [5, 11, 12]. It is well-known that a radical surgical therapy without neuromonitoring entails the risk of resecting accompanying healthy nerve fibers, which can lead to postoperative deficits and treatment-resistant pain [13, 14]. Therefore, it appears even more important to share own therapeutic approaches and experiences with this rare type of tumor. In this study, we retrospectively evaluated all patients suffering and surgically treated from a retroperitoneal nerve tumor in our neurosurgical department between 01/2002 and 12/2022, taking into account a combined visceral- and neurosurgical approach.

### Literature review, preoperative imaging and diagnosis

Considering the histomorphology of nerve tumors in the retroperitoneal space, most are BPNSTs, particularly schwannomas and neurofibromas [3]. This is consistent with our experience. 27 out of 34 histological examinations (79.4%) revealed either one of these entities, or a hybrid with elements of both tumors. By our knowledge, this is the largest series of retroperitoneal BPNSTs treated with a microsurgical technique [5, 13, 15]. Preoperatively, sufficient diagnostic is crucial for determining an appropriate treatment plan. According to the latest AWMF guidelines for peripheral nerve tumors, neurophysiological examination plays an essential role in the care of patients with nerve tumors, both for diagnosis and assessing prognosis in disease progression [16]. Imaging typically involves CT scans, ultrasound and MRI. Although characteristics like inhomogeneity, necrotic areas and irregular borders of the tumor can be helpful, imaging alone often is not reliable enough in differentiating between benign and malignant growth so a surgical and histological examination are essential [3]. BPNSTs mostly require a nerve-sparing surgical enucleation as demonstrated in Fig. 1. Hybrid nerve tumors show combined features of more than one tumor entity with schwannoma / neurofibroma and schwannoma / perineurioma being the most common combination [17]. The former was diagnosed twice in our patient collective. By use of intraoperative electrical stimulation, fascicle-free sections can be identified firstly, after which the tumor content can be obtained while sparing the nerve-bearing capsule [14]. In cases of perineuriomas, biopsy of enlarged nonfunctional fascicles mostly is the method of choice. A resection can lead to permanent neurological damage and mostly is not indicated due to lack of growth tendency. However, as a symptomatic treatment, an epineuriotomy can be performed to decompress the hypertrophic fascicle, which in our cases led to improvement of radicular pain [13]. Malignant nerve tumors have to be treated more radically. It is well known that patients with MPNSTs have a poor prognosis and survival, with NF1 being considered a negative prognostic factor. The success of therapy largely depends on adequate resection and rapid histological evaluation [12]. The issue of using a needle biopsy for diagnosing intermediate forms of nerve tumors is addressed in the current guideline for treatment of adult soft tissue sarcomas. Intermediate forms often contain both benign and potentially malignant components. A biopsy may miss the malignant portions, leading to an incorrect grading [16]. A good example of the challenges in diagnosis, even with an open resection, can be seen in case of a NF1 patient with a WHO grade 1 neurofibroma and rapid recurrence with necessity for reoperation. Considering the circumstance that NF1 patients often suffer from increased risk of malignancies, the presence of malignant precursors or components of a low-grade MPNST (that could not be captured histopathologically) cannot be dismissed.

### Surgical approaches to the retroperitoneal space

**Fig. 4:**
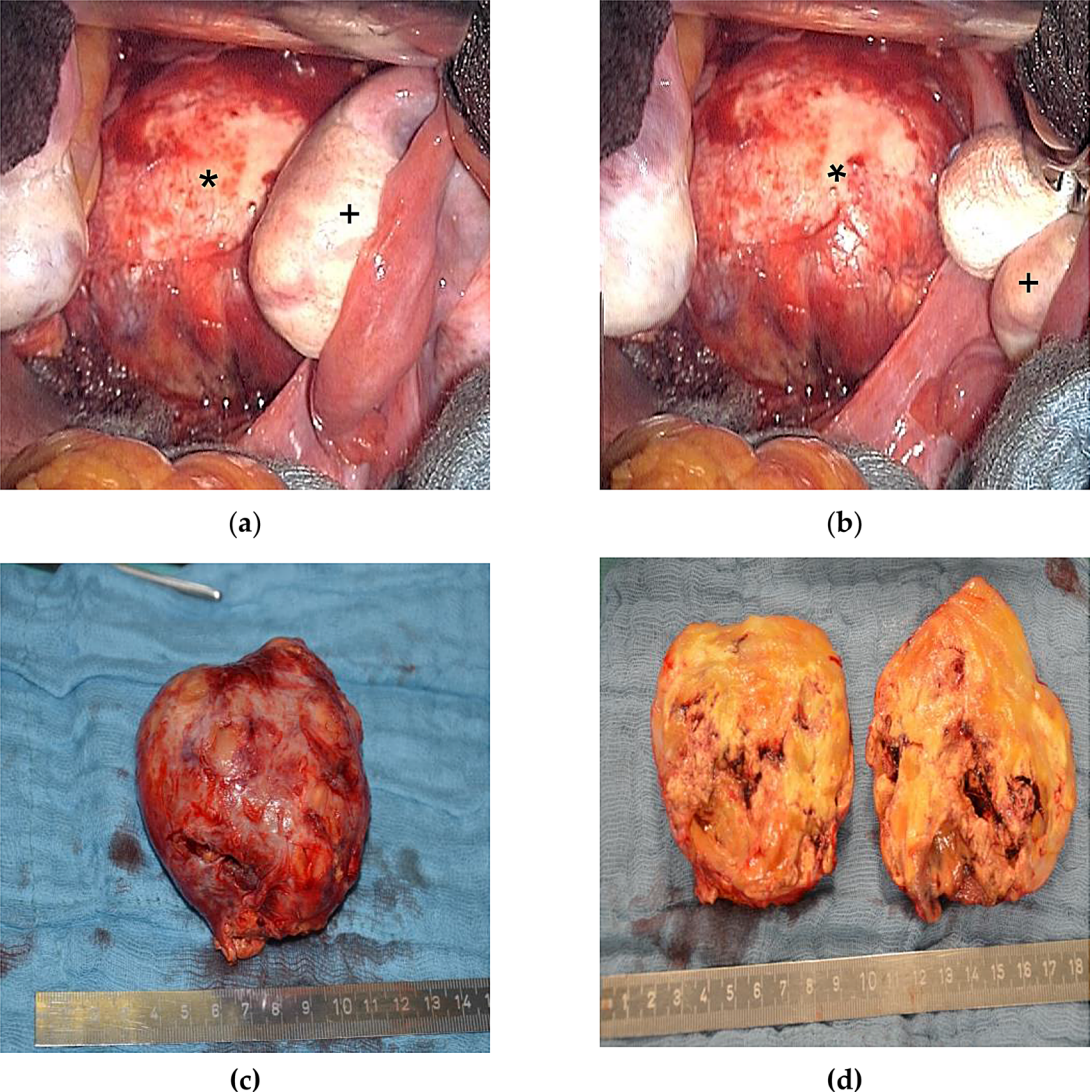
Depicted is a space-occupying presacral schwannoma with diameter of 8,5 cm of a 44-year-old female patient (**a**). After median lower abdominal laparotomy, the mass is carefully preparated after mobilizing the left compressed ovary (+) in direct proximity to the tumor (*). Ultimately, enucleation and complete removal of the mass was performed. The heterogeneous morphology of the benign tumor is clearly visible after sectioning (**c** / **d**). Patient described abdominal pain and bladder dysfunction preoperatively which disappeared in the postoperative follow up period

Resection can be achieved through either a ventral (trans- or extraperitoneal) or dorsal (retroperitoneal) approach. A median lower abdominal laparotomy allows good visualization of the surrounding organs and neuronal structures (Fig. 1c and d), while preserving vascular supply, particularly for tumors in the lower retroperitoneum [18, 19]. The pararectal approach can be used as an alternative to the conventional median laparotomy, particularly for tumors affecting the lumbosacral spine [20]. The dorsal approach, such as via a flank incision, is often used for tumors in the middle, dorsal retroperitoneum and near the spine, offering less invasiveness and sparing of the ventral abdominal organs [21]. In recent years, laparoscopic procedures for treatment of retroperitoneal nerve tumors have gained relevance. Several studies highlight the benefits of laparoscopic nerve surgery, including reduced invasiveness, shorter hospitalization rates, decreased need for pain medication and low intraoperative blood loss [22–24]. In a recent study conducted by a urological research group, 27 patients suffering from various retroperitoneal nerve tumors were treated using a retroperitoneoscopic approach. The results demonstrated a complete resection, a low mean operative time (98.3 ± 12.8 min), a short postoperative hospital stay (2.9 ± 1.0 days), and no recurrence during follow-up. In 4 cases, injuries to major blood vessels or the peritoneum were reported [25]. The limited working space and restricted maneuverability of laparoscopic instruments may make it more difficult to effectively handle and manipulate large retroperitoneal tumors (Fig. 4). In the mentioned urological study, all tumors were under 5 cm in size. Moreover, postoperative scarring may compromise adequate visibility, particularly in cases of recurrence or previous intra-abdominal surgery.

Although neurophysiological monitoring including intraoperative electrostimulation of nerves is not yet standard in laparoscopic and robot-assisted procedures, the technology is already feasible [26, 27]. Given the advantages of minimally invasive surgery, the further development of neurophysiological techniques and neuromonitoring in laparoscopic and robotic approaches within the retroperitoneum—where multiple nerval structures lie in close proximity—should be a priority for both surgeons and the industry.

Overall, there are indications that for benign and well-defined tumors, open and laparoscopic procedures could be equally effective in terms of complication and recurrence rate [23]. The chosen approach depends on the anatomical location, patient history (e.g. previous abdominal surgeries), the surgeon’s preference and personal experience [19, 28].

### Comparison of non-neurosurgical procedures and value of the combined surgical strategy

The lack of comparable, particularly monodisciplinary studies focusing on the neurological outcome, makes it challenging to do definitive statements regarding the advantages of the chosen procedure. However, several retrospective studies and case reports focusing on retroperitoneal nerve tumors from non-neurosurgical departments have been published (Table 2). In a retrospective analysis of 82 cases with retroperitoneal schwannomas between 1951 and 2007 conducted by a hepatobiliary surgery department, Li et al. reported a correct preoperative diagnosis in only 15.9% and a complete resection in 73.2% of all cases [29]. An increased recurrence rate was not observed and neurological outcomes not assessed. In a more recent retrospective study from 2022 by another visceral surgery center and 67 surgically treated retroperitoneal schwannomas, 92.5% of tumors were fully and 7.5% subtotal removed with a slow progression afterwards. Postoperative complications, including postoperative bleeding, paralysis of the lower extremities, or urinary fistula, occurred in 9,0% of all cases [30].

In a study from a gynecological department involving 45 patients with retroperitoneal schwannomas, a total resection was achieved in 82.2% of cases. In 9 / 45 patients, the surgery became more complex due to the tumor’s relationship with blood vessels or nerve roots, necessitating the involvement of general surgeons or neurosurgeons. Postoperatively, no lower limb discomfort was reported, and no recurrences were observed in the follow-up data [31].

**Table 2:** Non-neurosurgical case series of retroperitoneal nerve tumors

Source Discipline	Number of patients	Histology	Tumor size	Blood loss	ND	Complete removal	Recurrence rate
Li (2007) [29] Abdominal surgery	82 (1951–2004)	S (100%)	3–22 cm	N.R. (2500 ml in one case)	1.2% quadriceps femoris paralysis	73.2%	2.4% 63 months follow-up (Md)
Tang (2022) [30] Abdominal surgery	67 (2015–2021)	S (100%)	2.5–26 cm	20-9000 ml (300 ml Md)	3% paralysis and discomfort of the lower extremities	92.5%	7.5% 3–68 months follow-up (62 patients)
Goh (2005) [32] Abdominal surgery	5 (1989–2004)	S (100%)	4–14 cm	N.R.	N.R.	100%	0% 17 months follow-up (M)
Theodosopoulos (2008) [33] Abdominal surgery	5 (1991–2006)	S (100%)	7–20 cm	N.R.	20% hypoesthesia and weakness of the leg	80%	0% 37 months follow-up (Md)
Strauss (2011) [2] Abdominal surgery	28 (2001–2009)	S (100%)	5–23 cm	N.R.	N.R.	85%	0% 39 months follow-up (M)
Xiao (2021) [34] Abdominal surgery	30 (2012–2019)	G (100%)	2.8–15.6 cm	N.R.	N.R.	76.7%	0% 15.8 months follow-up (M) (26 patients)
Guz (1989) [35] Urology	6 (1984–1989)	S (50%) N (50%)	N.R.	N.R.	N.R.	100%	0% 12.6 months follow-up (M)
Cury (2007) [36] Urology	3 N.R.	S (100%)	3–12.5 cm	N.R.	N.R.	66.7%	0% 140 months follow-up (M)
Zhang (2016) [25] Urology	27 (2009–2016)	G (44.5%) S (37%) PG (18.5%)	2.8–4.4 cm	N.R. (blood transfusion in 5 cases)	N.R.	100%	0% 12–72 months follow-up
Yi (2021) [37] Gynecology	1	S (100%)	7 cm	600 ml	100% sensomotoric deficits of the leg and foot	100%	0% 24 months follow-up
Dede (2003) [38] Gynecology	3 N.R.	S (100%)	6–8 cm	N.R.	N.R.	100%	0% 18 months follow-up (M)

*Abbreviations* ND: Neurologic deterioration after surgery; Md: median; M: mean; S: schwannoma; N: neurofibroma; G: ganglioneuroma; PG: paraganglioma

In our presented cases of BPNSTs, the chosen visceral- / neurosurgical approach demonstrated favorable outcomes. Except for one case of a neurofibroma, follow-up examinations over a median of 24 months indicated no instances of tumor recurrence in BPNSTs. In the mentioned case, a repeat surgical intervention was indicated due to recurrence. Re-resection of both progressive MPNSTs was not considered. Out of all treated patients, only three reported persistent pain, suggesting a high success rate in this regard. Our results are consistent with previous studies conducted by other research groups such as Hajiabadi et al. and Heinen et al., both of which describe the benefit of incorporating neurosurgical involvement in patient treatment [5, 13]. Considering that BPNSTs, such as schwannomas or neurofibromas, are the most common retroperitoneal nerve tumors, it is not surprising that these masses are often included in case series by non-neurosurgical teams (Table 2). Notably, there is no clear difference in residual tumor and recurrence rates when comparing outcomes between procedures with and without neurosurgical involvement. Regarding blood loss and duration of surgery, neither was found to be elevated in our study compared to non-neurosurgical and other case series [15, 31]. However, several case reports from non-neurosurgical departments, such as published by Yi et al. desribe serious neurological complications due to preoperative misjudgment of the diagnosis [37]. The absence of information regarding neurological outcomes could be associated with a significant number of unreported cases of sensory and motor deterioration. Therefore, an important aspect of the outcome is not only tumor-free status or the absence of recurrences in patients, but also good mobility and neurological integrity throughout the follow-up period. Considering that neither in the present study nor in other neurosurgical teams such as those by Hajiabadi et al. or Benato et al., reported any motor deterioration after surgical treatment of BPNSTs, it seems evident that the chosen methods and inclusion of neurosurgical expertise could be more than non-inferior to monodisciplinary approaches [5, 15]. Early neurosurgical involvement could prevent situations where a neurosurgeon is only consulted intraoperatively in acute situations or where patients are referred to neurosurgical departments postoperatively due to residual tumors for evaluation of re-operation [31, 33].

Multicenter prospective studies are urgently needed to address the issue of low case numbers and improve the assessment of various surgical approaches. In this regard, the fundamentally correct approach of interdisciplinary collaboration should extend beyond just monodisciplinary or combining neurosurgery and visceral surgery approaches. It should encompass a broad range of specialties involved in treating conditions within the retroperitoneal space.

## Data Availability

No datasets were generated or analysed during the current study.
